# The Antitumor Effects of Triterpenoid Saponins from the *Anemone flaccida* and the Underlying Mechanism

**DOI:** 10.1155/2013/517931

**Published:** 2013-09-26

**Authors:** Lin-Tao Han, Ying Fang, Ming-Ming Li, Hong-Bing Yang, Fang Huang

**Affiliations:** Key Laboratory of Traditional Chinese Medicine Resource and Compound Prescription of the Ministry of Education, Hubei University of Chinese Medicine, Wuhan 430065, China

## Abstract

*Anemone flaccida* Fr. Schmidt, a family of ancient hopanoids, have been used as traditional Asian herbs for the treatments of inflammation and convulsant diseases. Previous study on HeLa cells suggested that triterpenoid saponins from *Anemone flaccida* Fr. Schmidt may have potential antitumor effect due to their apoptotic activities. Here, we confirmed the apoptotic activities of the following five triterpenoid saponins: glycoside St-I4a (1), glycoside St-J (2), anhuienoside E (3), hedera saponin B (4), and flaccidoside II (5) on human BEL-7402 and HepG2 hepatoma cell lines, as well as the model of HeLa cells treated with lipopolysaccharide (LPS). We found that COX-2/PGE2 signaling pathway, which plays key roles in the development of cancer, is involved in the antitumor activities of these saponins. These data provide the evidence that triterpenoid saponins can induce apoptosis via COX-2/PGE2 pathway, implying a preventive role of saponins from *Anemone flaccida* in tumor.

## 1. Introduction

 The genus *Anemone *comprises 150 species which are widely distributed around the world, and about 50 species of *Anemone *are found in China [1, 2]. Triterpenoid saponins are the characteristic components and also the main active ingredients of *Anemone* [3]. *Anemone flaccida *Fr. Schmidt, commonly known as “Di Wu,” belongs to the family Ranunculaceae which is widely used as a Chinese folk medicine. Several triterpenoid saponins were isolated, and their antitumor activities have been investigated [4]. In our previous work, the following five triterpenoid saponins have been isolated from the plant *Anemone flaccida*: hedera saponin St-I4a (1), hedera saponin St-J (2), anhuienoside E (3), hedera saponin B (4), and flaccidoside II (5) [5]. The growth inhibition effects and apoptosis-induction activities of these compounds have also been identified in HeLa cells, while the molecular mechanisms remain unknown.

 The COX-2/PGE2 pathway is considered to play a crucial role in the development of cancer [6, 7]. cyclo-oxygenase 2 (COX-2), an inducible form of the enzyme that catalyses the first step in the synthesis of prostanoids from arachidonic acid, is involved in inflammatory diseases and carcinogenesis. It has been proved that COX-2 is upregulated in multiple types of tumors, including carcinomas of the lung, breast, colon, prostate, and urinary bladder in human and animal models [8, 9]. COX-2, which may promote angiogenesis and so drive the malignant phenotype, has been identified as a possible early diagnostic marker of virus-associated human malignant neoplasms [10]. Alterations to cyclooxygenase-2 (COX-2) expression and the abundance of its enzymatic product prostaglandin E2 (PGE2) influence the genesis and development of colorectal cancer [11]. Multiple distinct mechanisms of downregulation of the COX-2/PGE2 pathway in tumor genesis have been identified: promoting tumor maintenance and progression, as well as encouraging metastatic spread. Studies in different cancers have suggested that several downstream proangiogenic growth factors can be increased by COX-2, like VEGF and Flt-1, Flk-1/KDR, angiopoietin-1, tie-2, MMP2, and OPN. The pro-inflammatory mediator prostaglandin E2 (PGE2) has been incriminated as a major COX product involved in tumor development and progression [12–14]. COX-2 promotes the release of PGE2, which acts on its cell surface G protein-coupled receptors, the E series of prostaglandin (EP) receptors, designated as EP1 to EP4 [15]. However, no lires of evidence have been reported on the modulation of COX-2 expression in tumor cells by triterpenoid saponins of *Anemone* and whether the COX-2/PGE2 pathway is involved in the anti-tumor activities of these saponins remains unknown.

 In the present study, we show that total saponin (TS) and five oleanane type triterpenoid saponins which are isolated from the rhizome of *A. flaccida*, inhibit cell growth, and also induce apoptosis on several cancer cell lines including hepatic tumor (HepG2 and BEL-7402), as well as the model of cervical tumor cells (HeLa) treated with lipopolysaccharide (LPS), which provide further evidence for our previous data. Meanwhile, we confirm that the expression of COX-2 is inhibited by performing these saponins. These results implied that the triterpenoid saponins possess potential anti-tumor activities and they exert their inhibition effects via inhibiting COX-2/PGE2 pathway.

## 2. Materials and Methods

### 2.1. Plant Material and Reagents

 Rhizome of *A. flaccida* Fr. Schmidt was collected from Jiufeng County of Hubei Province, China, as mentioned in our previous work. Total saponins (TS) and single triterpenoid saponins 1–5 were isolated from *A. flaccida *by our research group, and their relative contents were determined by using normalization method of chromatographic peak areas. All of them were dissolved in dimethyl sulfoxide (DMSO) and diluted in PBS for storage in −20°C freezer which is used in all subsequent experiments. Dulbecco's modified Eagle medium (DMEM) was purchased from Gibco (Grand Island, NY, USA). The fetal calf serum (FCS), dimethyl sulfoxide (DMSO), 3-[4,5-dimethylthiazol-2-yl]-2,5-diphenyltetrazolium bromide (MTT), and lipopolysaccharide (LPS) were obtained from Sigma-Aldrich (St. Louis, MO, USA). Rabbit polyclonal antibody (pAb) COX-2 against cyclooxygenase 2 (ab6665) and goat polyclonal to beta Actin antibody (ab8229) were purchased from Abcam Inc. (Cambridge, MA). HRP-conjugated anti-rabbit or anti-goat secondary antibodies, chemiluminescent substrate kit, and phosphocellulose units were from Pierce Chemical Company (Rockford, IL, USA).

### 2.2. Cell Culture and Treatment

 Human hepatocellular liver carcinoma (HepG2) cell line was purchased from American Type Culture Collection (Manassas, VA). HepG2 cells were grown in alpha-MEM supplemented with 10% FBS and 2 mM L-glutamine. Human hepatoma cell line BEL-7402 was purchased from Cell Research Center of Chinese Academy of Science (Shanghai, China). BEL-7402 cells were cultured in RPMI-1640 medium with L-glutamine supplemented with 10% fetal calf serum and 1% antibiotic-antimycotic solution (Gibco, Carlsbad, CA), at 37°C in a humidified atmosphere of 5% CO_2_ in air. The Human cervix epithelial carcinoma (HeLa) cell line was obtained from China Center for Type Culture Collection. HeLa cells were routinely cultured in Dulbecco's modified Eagle medium (DMEM) supplemented with 1% nonessential amino acids, 10% fetal calf serum, penicillin (100 U·mL^−1^), and streptomycin (100 mg·mL^−1^) in humidified air with 5% CO_2_ at 37°C. Monolayers of HeLa cells were treated with 50 mg/mL lipopolysaccharide (LPS) for 24 h.

### 2.3. Assessment of Cell Proliferation by MTT Assay

 All cancer cells as mentioned were seeded in 96-well plates at an initial cell density of 5 × 10^4^ cells/cm^2^ and then individually treated with TS at the following gradient concentrations: 40.0, 20.0, 10.0, 5.0, and 2.50 *μ*g*·*mL^−1^ or single triterpenoid saponins 1–5 at the following gradient concentrations: 40.0, 20.0, 10.0, 5.0, and 2.50 *μ*mol·L^−1^. After incubation for 24 h, 20 *μ*L MTT (5 mg·mL^−1^, Sigma, USA) was added to the culture medium and incubated for an additional 4 h at 37°C. After careful removal of culture medium, 150 *μ*L DMSO was loaded to each well to coloration. The plates were shaken vigorously for 30 min to ensure complete solubilization at room temperature; the absorbances were determined with a multiwell spectrophotometer (Bio-Rad, Hercules, CA, USA) at the wavelength of 490 nm. The growth inhibitory ratio was defined as the percentage of differences between negative control and treated group. The following formula was used: inhibitory ratio of growth (%) = (1 – (average absorbance of treated group/average absorbance of control group)) × 100%. According to the analysis of the inhibitory ratio of growth versus concentration, the 50% inhibitory concentrations of cells (IC_50_) were calculated with software SPSS Version 16.0 (SPSS Inc., Chicago, USA).

### 2.4. Evaluation of Apoptosis by Annexin V FITC/PI Double-Staining Assay and Flow Cytometry Analysis

Apoptosis was assayed by using an Annexin V-PI staining kit by following the manufacturer's procedure (KeyGen Biotech. Co. Ltd, Nanjing, China). The apoptotic rate was automatically quantified by flow cytometry with the standardized program of the instrument (FACSCalibur, BD Biosciences, San Jose, CA). All cancer cells were treated with 5.0 mg·L^−1^ TS or 5.0 *μ*mol·L^−1^ single triterpenoid saponins 1–5 individually for 24 h, and then harvested by trypsinization, washed twice with ice-cold PBS, then double-stained by Annexin-V FITC and PI according to the instruction. Then, the apoptotic rate was determined by FACScan flow cytometry. The cells in the FITC positive and PI-negative fraction were regarded as apoptotic cells.

### 2.5. RNA Isolation and Reverse Transcription (RT)

 Cells were seeded and grown in a manner analogous to that described above. After 24 h exposure to TS or single triterpenoid saponins 1–5, cells were washed with PBS. The extractions of total cellular RNA of all kinds of cancer cells were conducted using Trizol Reagent (Invitrogen, Carlsbad, CA, USA) according to the supplier's instructions. After RNA isolation, cDNAs were formed by using reverse transcription kit (Toyobo, Osaka, Japan) according to the manufacturer's protocol. RT products were aliquoted in equal volumes and stored at −20°C.

### 2.6. Evaluation of mRNA Expression of COX-2 by Quantitative Real-Time Polymerase Chain Reaction

 The real-time PCR (RT-PCR) was used to evaluate mRNA expression of COX-2 in cancer cells after treatment with TS or single triterpenoid saponins 1–5. Each 25 *μ*L reaction contained 12.5 *μ*L SYBR Green real-time PCR master mix (Toyobo, Osaka, Japan), 2.5 *μ*L cDNA (10x diluted), 2.5 *μ*L plus solution, 2 *μ*L (5 pmol/*μ*L) primer, and 5.5 *μ*L ddH_2_O. The real-time PCR specific primers were 5′-ACGCTCAGGAAATAGAAA CCG-3′ (forward) and 5′-GTCAACGTCAAGGAGTCGCAG-3′ (reverse) for COX-2, 5′-CAA CTCCATCATG AAGTGTA A-3′ (forward) and 5-CCACACGGAGTACTT GCGCTG-3′ (reverse) for *β*-actin. The PCR amplification was performed under the following conditions: 40 cycles of denaturation at 95°C for 15 s, annealing at 60°C for 15 s, and extention at 72°C for 45 s, using the real-time PCR detection system (HongShi, ShangHai, China). The experiment was performed three times to achieve reproducibility. The *β*-actin gene was used as an internal reference to normalize the expression of COX-2. The mean value of the replicates for each sample was calculated and expressed as the threshold cycle (Ct). Relative quantification was determined by the 2-ΔΔCt method, and fold differences were calculated as previously described.

### 2.7. Western Blotting

 Cells were individually treated with 5.0 mg·L^−1^ TS or 5.0 *μ*mol·L^−1^ single triterpenoid saponins 1–5 for 24 h. Then, cell lysates were prepared for western blotting analysis of COX-2 by using whole cellular protein extraction kits (Santa Cruz Biotechnology, Santa Cruz, CA), and protein concentrations were quantified using a Bio-Rad protein assay (Bio-Rad, Hercules, CA). The cell lysates with equal amount of proteins from each group (i.e., 50 *μ*g for COX-2 and 10 *μ*g for DM1A) were firstly separated by 10% SDS-polyacrylamide gel electrophoresis (PAGE) and then electrically transferred to polyvinylidene fluoride membrane (Merck Millipore, USA). Membranes were then blocked in 5% powdered nonfat milk in TBS solution for 1 h. COX-2 was probed with primary antibody COX-2 (1 : 500), *β*-actin was probed with anti-*β*-actin specific rabbit polyclonal IgG primary antibody (Santa Cruz Biotechnology, Santa Cruz, USA) (1 : 500) at 37°C for 2 h. After washing three times by TBS/0.2% Tween, the membranes were then covered with an HRP conjugated goat anti-rabbit secondary IgG antibody (1 : 2000 dilution) for 1 h at room temperature. Immunoreactive bands were then visualized by enhanced chemiluminescent substrate kit and exposed to CL-XPosure film. Protein bands were quantitatively analyzed by Kodak Digital Science 1D software (Eastman Kodak Company, New Haven, CT, USA) and were expressed as sum optical density.

### 2.8. Determination of PGE2 Production

 Prostaglandin E2 (PGE2) is a major product of COX-2 catalytic arachidonic acid. PGE2 levels were determined in order to estimate COX-2 activity. To quantify the levels of PGE2 released into culture medium, competitive enzyme-linked immunosorbent assay (ELISA) kit was performed according to the manufacturer's instructions (R&D Systems Inc. USA) and the absorbance was determined with a multiwell spectrophotometer (Bio-Rad, Hercules, CA, USA) at wavelength 450 nm.

### 2.9. Statistical Analysis

Data were analyzed using SPSS 12.0 statistical software (SPSS Inc., Chicago, Illinois, USA). Student's *t*-test was used to determine the statistical significance of differences. All date were presented as the means ± standard deviation (SD) of at least three independent experiments. Statistical significance level was *P* < 0.05.

## 3. Results

### 3.1. Total Saponin (TS) and Triterpenoid Saponins 1–5 Inhibit the Proliferation of Cancer Cells

**Figure 1:**
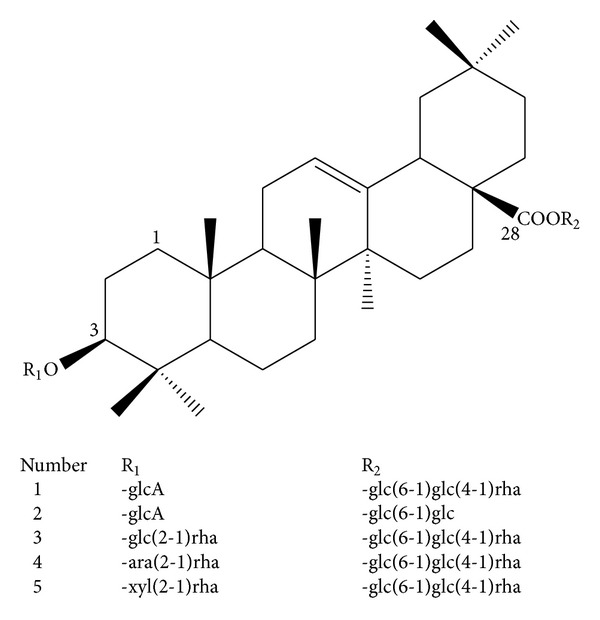
Structures of compounds 1–5.

 Total saponins (TS) and single triterpenoid saponins 1–5 were isolated from *A. flaccidas *and their relative contents are >98% determined by using normalization method of chromatographic peak areas. Our previous work has shown the structures of compounds 1–5 (Figure 1) and proved that single triterpenoid saponins 1–5 from *Anemone flaccida *induce apoptosis activity in HeLa cells [5]. To further investigate the effects of total saponin (TS) and triterpenoid saponins 1–5 on the proliferation of cancer cells, a panel of three human cancer cell lines, including the human cervix carcinoma cell model (High COX-2 expressed HeLa cells induced by LPS treatment) and two human hepatocellular carcinoma cell lines (BEL-7402 and HepG2) were used in the present study. All cancer cells were individually treated with TS at the following gradient concentrations: 40.0, 20.0, 10.0, 5.0, and 2.50 *μ*g·mL^−1^ or single triterpenoid saponins 1–5 at the following gradient concentrations: 40.0, 20.0, 10.0, 5.0, 2.50, *μ*mol·L^−1^. After incubation for 24 h, cell viabilities were tested by MTT. We have found that TS and single triterpenoid saponins 1–5 showed dose-dependent growth inhibitions in all cell lines mentioned above, and cancer cells treated with 40.0 mg·L^−1^ TS or 40.0 *μ*mol·L^−1^ single triterpenoid saponins 1–5 can achieve the best inhibition rates (data not shown). Although all of these compounds performed antiproliferative effects on indicated cell lines, flaccidoside II (single triterpenoid saponin 5) has shown the most significant inhibitory activities on BEL-7402 and HeLa/LPS cells (Table 1). Hedera saponin B (4) also exhibited moderate inhibitory, while anhuienoside E (3) revealed middle inhibitory activities except the lowest inhibition rate on BEL-7402 cells. These data suggest that total saponin and triterpenoid saponins 1–5 inhibit the proliferation of cancer cells. 

### 3.2. Total Saponin and Triterpenoid Saponins 1–5 Facilitate Apoptosis of Cancer Cells

**Figure 2:**
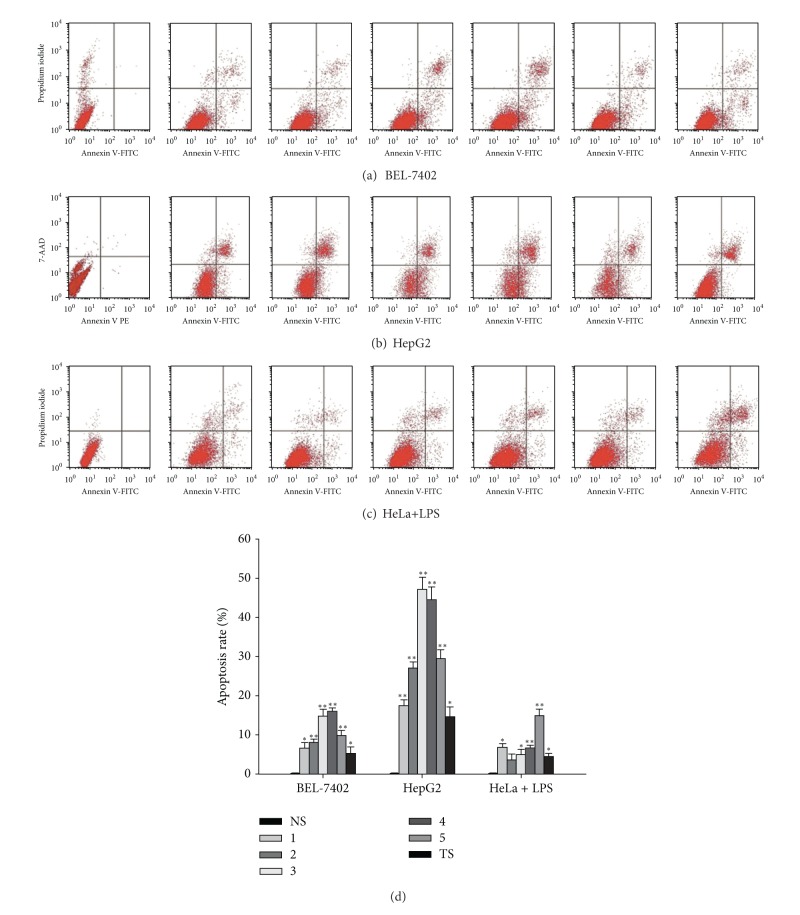
Effects of TS and triterpenoid saponins 1–5 on apoptosis rate of cancer cells. BEL-7402 ((a), (d)) and HepG2 cells ((b), (d)), as well as high COX-2 expressed HeLa cells induced by LPS treatment ((c), (d)), were then treated with 40.0 *μ*g·mL^−1^ TS or 40.0 *μ*mol·L^−1^ single triterpenoid saponins 1–5, respectively, for 24 h, and the apoptotic rate was determined by Annexin V-PI staining and flow cytometry for quantification. *P < 0.05, **P < 0.01 versus control (mean ± SD).

 One possible cause for inhibition of the proliferation of cancer cells could be the increased apoptosis. Caspase-3 is a well-known caspase protein which is situated at pivotal junctions in apoptosis pathways. Both extrinsic (death ligand) and intrinsic (mitochondrial) pathways activate caspase-3 by proteolytic cleavage, and caspase-3 then cleaves vital cellular proteins or other caspases as an executioner caspase [16]. The significance of dysfunction of caspase-3 mediated apoptotic pathway in tumor progression has been widely reported and greatly expanded [17, 18]. It has been reported by our group that TS and single triterpenoid saponins 1–5 of *A. flaccida* perform anticancer effects through caspase-3 mediated apoptotic pathway on HeLa cell line. To further confirm the proapoptotic effects of TS and single triterpenoid saponins 1–5, here we detect whether they inhibit cell growth in other cancer cell lines though inducing apoptosis by using flow cytometry to evaluate apoptosis rate. As shown in Figures 2(a)–2(d), the increased apoptosis rates were observed in cancer cells which were treated with TS and single saponins 1–5. It is noteworthy that the rank of proapoptotic effects of single saponins were observed as 5 > 1 > 4 > 3 > 2 in high COX-2 expressed HeLa cells induced by LPS, while the order of which as 5 > 3 > 4 > 1 > 2 was detected in HeLa cells in our previous work.

 Interestingly, we have also found that sharp apoptosis was induced by compound 4 in BEL-7402 cell line; meanwhile, compound 3 facilitates dramatic apoptosis in HepG2 cell line, as shown in Figure 2. These data imply the caspase-3 mediated apoptotic pathway is involved in anticancer activities of TS and saponins 1–5. 

### 3.3. Total Saponin and Triterpenoid Saponins Inhibit the COX-2/PGE2 Pathway in Cancer Cells

**Figure 3:**
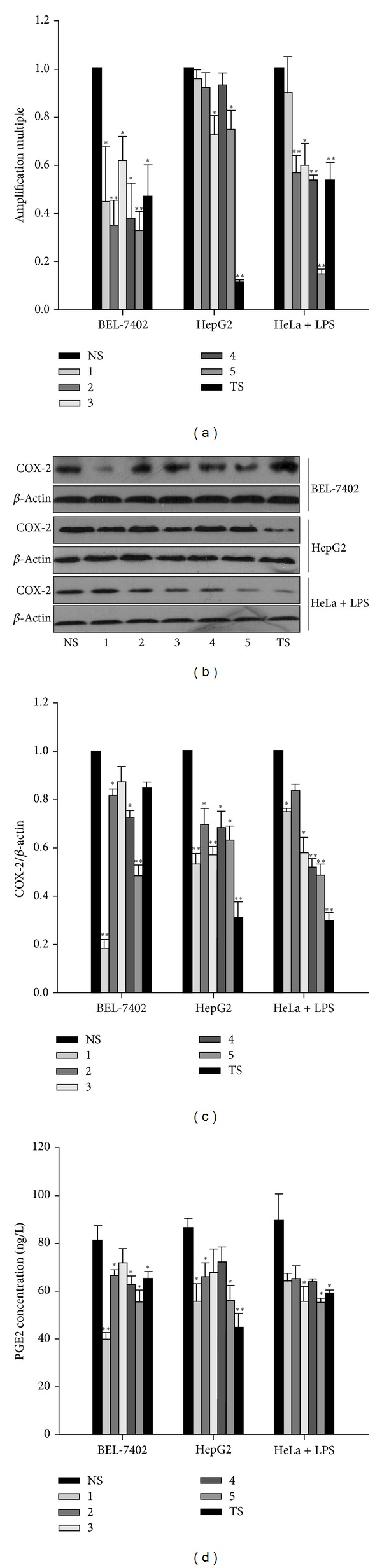
Effects of TS and triterpenoid saponins 1–5 on the COX-2/PGE2 pathway in cancer cells. ((a)-(b)) BEL-7402 and HepG2 cells, as well as high COX-2 expressed HeLa cells induced by LPS treatment, were then treated with 40.0 *μ*g·mL^−1^ TS or 40.0 *μ*mol·L^−1^ single triterpenoid saponins 1–5, respectively, for 24 h. The COX-2 mRNA level (a) was measured by quantitative real-time PCR assay and the COX-2 protein level ((b)-(c)) was measured by western blotting, which was normalized by *β*-actin. (c) Quantitative analysis of the blots in (b). (d) Effects of TS and single saponins 1–5 on PGE2 synthesis level were assessed by Elisa. *P < 0.05, **P < 0.01 versus control (mean ± SD).

 Increased levels of COX-2, which is regarded as a play-marker of tumor angiogenesis, and its product PGE2 have been identified in cancer cells [19]. To investigate whether TS and single saponins 1–5 from *A. flaccida *can inhibit COX-2/PGE2 pathway which may be involved in their proapoptotic effects on cancer cells, we first measured COX-2 mRNA with or without TS and triterpenoid saponins treatments in cancer cells by performing quantitative real-time PCR assay and also evaluated COX-2 expression in tumor cells at the protein level using western blot. We have found that TS and single saponins 1–5 significantly suppress COX-2 on both mRNA (Figure 3(a)) and protein levels (Figures 3(b)-3(c)) in all cancer cells compared with control groups, as shown in Figure 3. In particular, we have noticed that triterpenoid saponin 5 (Flaccidoside II) has more intensive inhibiting effect on COX-2 mRNA expression compared with TS and other triterpenoid saponins (Figure 3(a)), while all saponins have different effects on different cancer cells. More interestingly, the dramatic inhibiting effect of TS on COX-2 protein expression has also been observed as well as mRNA expression level in different cell models except Bel-7402 cell line (Figures 3(b)-3(c)). Then, we further assessed the effects of TS and single saponins 1–5 on PGE2 synthesis level, the product of COX-2. As expected, we observed declines on PGE2 production in cancer cells after treatments with TS or single saponins 1–5, as shown in Figure 3(d). These data implied that TS and triterpenoid saponins 1–5 inhibit the COX-2/PGE2 pathway in cancer cells which may be one of the mechanisms of their apoptotic effects.

## 4. Discussion

*Anemone flaccida *has been reported to have a broad spectrum of biological activities, including anti-inflammatory, anticonvulsant, and anticancer [3, 20]. We have reported that natural triterpenoid saponins, including glycoside St-I4a (1), glycoside St-J (2), anhuienoside E (3), hedera saponin B (4), and flaccidoside II (5), induce apoptosis in HeLa cells [5]. Since interest in natural compounds for development of novel, clinically useful anticancer agents has grown in recent years [21], here we further validate the potential antitumor effect of them in cervical cancer cells and hepatocellular carcinoma cells. In our present work, we find that TS and single triterpenoid saponins 1–5 from *A. flaccida* inhibit proliferation and facilitate apoptosis on high COX-2 expressed HeLa cells induced by LPS treatment, BEL-7402, and HepG2 cells. 

 Overexpression of cyclooxygenase-2 (COX-2) and the abundance of its enzymatic product prostaglandin E2 (PGE2) have key roles in development of colorectal cancer [22]. Suppression the expression of COX-2 is an attractive approach to target cancers which show upregulated COX-2. However the modulation of COX-2/PGE2 pathway in tumor cells by triterpenoid saponins of *Anemone *is not reported. In the present study, we observed that TS and single triterpenoid saponins 1–5 from *A. flaccida* facilitate apoptosis, and COX-2/PGE2 signaling pathway is regulated in hepatocellular carcinoma cells and cervical cancer cells, which might be one of the mechanisms of their antitumor activities. Based on the chemical structure of compounds (Figure 1), these five triterpenoid saponins have the same sapogenin and oleanolic acid, which can be classified into two groups: compounds 1 and 2 take the form of group I, in which a glucuronic acid link to the position of C-3; while compounds 3–5 take the form of group II, in which an L-rhamnopyranose links to other sugars at the end of glycosidic chain in C-3 position. Here, we find that compounds 3–5 show higher apoptosis-facilitating activities compared with compounds 1 and 2 in our presented cancer cells, which might be due to the glycosidic chain's polarity of C-3 position. We also compare the apoptotic effects of these compounds on high COX-2 expressed HeLa cells induced by LPS treatment with their effects on HeLa cells with basal COX-2 expression which have been shown in our previous work; compounds 1 and 5 show much higher sensitivities to facilitate apoptosis when COX-2 is overexpressed, while effects of compounds 2–4 and TS do not show significant differences in HeLa cells with or without LPS treatment. These data give us a hint that compound 5 could be the best candidate for chemoprevention studies to target cancers with overexpressed COX-2 compared with others in our present work. 

 Furthermore, we have shown that TS and triterpenoid saponins inhibit COX-2 expression in cancer cell models on both mRNA and protein levels and declines on PGE2 production in cancer cells after treatment with TS or single saponins 1–5 were also observed. Interestingly, we have found that TS and single saponin 5 sharply reduced PGE2 levels in LPS-stimulated HeLa cells while causing minor reduction in PGE-2 levels in the unstimulated condition [5], which hint that they are more effective in the case when COX-2 is highly expressed. Other researches have also shown inhibition of tumor proliferation, survival, and metastasis by pentacyclic triterpenoids [23, 24]; our data not only verify the potential role of oleanane-type triterpene saponins in prevention and therapy of cancer specifically in hepatocellular carcinoma cells via the inhibition on the COX-2/PGE2 pathway but also show that triterpene saponins investigated in the present work have higher apoptotic activities verified by lower IC50 value which might be due to the C-3 position glycosidic chain's polarity [25]. 

## 5. Conclusion

**Figure 4:**
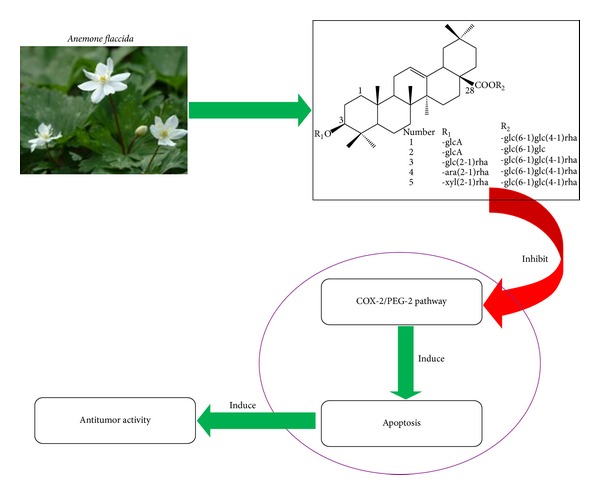
A model for the antitumor effects of triterpenoid saponins from the *Anemone Flaccida* and the underlying mechanism. TS and triterpenoid saponins 1–5 which are isolated from *A. flaccida *Fr. Schmidt have significant anticancer activities in cancer cell lines, and their induction on apoptosis via COX-2/PGE2 pathway is involved in the underlying mechanisms.

 In summary, our present study demonstrated that TS and triterpenoid saponins 1–5 which are isolated from *A. flaccida *Fr. Schmidt have significant anticancer activities *in vitro*, and their facilitations on apoptosis via COX-2/PGE2 pathway are involved in the underlying mechanisms (Figure 4).

## Figures and Tables

**Table 1:** Summary of  IC_50_ values of cancer cell lines as mentioned after 40.0 *μ*mol·L^−1^  single triterpenoid saponins 1–5 treatments. IC_50_ (*μ*mol·L^−1^).

Group of treatment	IC_50_ (*μ*mol·L^−1 ^)		
	HeLa + LPS	BEL-7402	HepG2
1	16.73	14.67	20.10
2	18.56	15.83	18.34
3	13.26	25.85	11.51
4	10.48	9.90	13.59
5	8.14	8.41	8.92

1: Hedera saponin St-I4a; 2: Hedera saponin St-J; 3: Anhuienoside E; 4: Hedera saponin; 5: Flaccidoside II.
